# Meeting abstracts from the Annual Conference “Clinical Genetics of Cancer 2024”

**DOI:** 10.1186/s13053-026-00330-5

**Published:** 2026-03-05

Szczecin, Poland. 26^th^-27^th^ September 2024

Edited by Jan Lubiński

The Supplement Editor declares no competing interests

The International Conference ‘Clinical Genetics of Cancer 2024’ was supported by a grant from the polish Ministry of Science and Higher Education under the ‘Excellent Science II’ programme (project no. KONF/SP/0086/2024/02).

## A1 Axiom PolMut1 - polish custom genetic test for cancer predispositions and other genetically determined diseases

Katarzyna Białkowska^1^, Anna Jakubowska^1,2^

^1^International Hereditary Cancer Center, Department of Genetics and Pathology, Pomeranian Medical University in Szczecin, Szczecin, Poland; ^2^Independent Laboratory of Molecular Biology and Genetic Diagnostics, Pomeranian Medical University in Szczecin, Szczecin, Poland

The aim of the study was to develop a unique genetic test for the Polish population that would enable the detection of all known mutations associated with an increased predisposition to cancer and other genetically determined non-cancerous diseases. The test needed to be cost-effective, rapid, and widely accessible.

Based on available literature data and the results of our previous studies on high hereditary predisposition to cancer, a database of genetic variants (so-called genetic markers) present in the Polish population and linked to a predisposition to cancer and other non-cancerous diseases was created. Ultimately, 2,136 genetic markers were approved for genotyping after thorough scientific evaluation and technological consultation. These markers included pathogenic, likely pathogenic, and VUS (variants of uncertain significance) variants in cancer-predisposing genes, non-cancer disease genes, as well as variants in microelement-metabolism genes, and markers for the breast cancer polygenic risk score (PRS 313).

In the subsequent step, microarrays (under the acronym PolMut1; Thermo Fisher Scientific) containing the selected genetic markers were designed and manufactured, followed by large-scale DNA genotyping. The PolMut1 microarray study included 5,376 genomic DNA samples. Among the genotyped samples, 104 contained previously detected and confirmed pathogenic or likely pathogenic variants in one of the following cancer-predisposing genes: *ABRAXAS, ATM, BLM, BRCA1, BRCA2, BRIP1, CHEK2, MLH1, MSH2, MSH6, MUTYH, PALB2, PMS2, RAD50, RAD51C, STK11, TP53*, and *VHL*. These samples served as ‘positive’ controls for analysis using the PolMut1 microarray. Other samples were from individuals with a family history of cancer (HBC/HBOC/HOC, CFA, HNPCC, HPC, HSC, Familial MM syndromes), with approximately 3,800 individuals affected by cancer.

Quality control (QC) assessment revealed that genotyping using the PolMut1 microarray passed QC with a sample pass rate of 98.1% and an average QC call rate of 99.6% (thresholds were set at ≥95% and ≥98.5%, respectively). Results from genotyping ‘positive’ controls showed that the PolMut1 array confirmed 87% of the variants. The remaining 13% will be sequenced to investigate the reasons for non-compliance.

Additionally, we plan to verify all detected variants using Sanger sequencing, identify variants that may correlate with cancer incidence and other diseases, and calculate PRS 313 for the Polish population. Ultimately, we aim to establish whether analysis using the „Axiom PolMut1” array has scientific and diagnostic value.

### Funding

Research task carried out within the framework of the programme of the Minister of Science and Higher Education called ‘Regional Excellence Initiative’ in the years 2019–2022 project number 002/RID/2018/19 amount of funding 12 000 000 PLN.

## A2 Methylome-based Pan Cancer Classifier (MePCC) - pan-cancer classification based on methylomics data

Jan Bińkowski^1,2^, Tomasz K. Wojdacz^1,2^

^1^Independent Clinical Epigenetics Laboratory, Pomeranian Medical University, Szczecin, Poland; ^2^Regional Center of Digital Medicine; Pomeranian Medical University, Szczecin, Poland

**Keywords**: Epigenomics, Machine-learning, Classification, Cancer

### Funding

The human methylome undergoes extensive alterations during carcinogenesis. While some DNA methylation changes occurring during the neoplastic transformation are stochastic, acting similarly to the accumulation of passenger mutations, others are critical for cancer development. Methylome changes essential for neoplastic transformation are stably transmitted during cell division, making them potential candidates for diagnostic biomarkers. In this study, we investigated whether methylomics data obtained using the Illumina BeadChip array are sufficient to develop a highly accurate, machine-learning (ML) based pan-cancer classifier.

### Methods

To build the classifier we used methylation profiling data (Illumina BeadChip microarrays) from public repositories including Genomic Data Commons and Gene Expression Omnibus databases. Overall the dataset for the development of our classifier included 12 156 genome wide methylation profiles for 39 types of cancer and 11 healthy tissues. Following the generally accepted principles of development of ML-based solutions we randomly (using cluster-sampling strategy) divided the entire dataset into three independent subsets: training (n=7805 samples), testing (n=3264 samples), and validation (n=1087 samples). Three types of measurements: (1) methylation levels expressed as beta-values, (2) co-methylation levels expressed as delta-beta-values, and (3) CNVs inferred from normalized probe intensities, were extracted from each microarray. The new type of effect size metric adjusted for sample heterogeneity was used to select the microarray measurements that best discriminated between samples in training data set. We then trained, tuned, and evaluated seven commonly used ML models using nested cross-validation combined with grouped-stratified sampling. Additionally, to ensure the quality of predictions we implemented a local outlier factor algorithm to detect abnormal samples - anomalies (outliers or novelties) that could potentially affect the confidence of model predictions.

### Results

The logistic regression model achieved the best average specificity and sensitivity of pan-cancer classification in training set during the cross-validation procedure. Importantly, in the testing procedure model achieved an impressive average sensitivity and specificity of 0.94, and this level of sensitivity and specificity was also achieved using independent validation dataset, confirming excellent classification power of the classifier.

### Conclusions

We demonstrated that despite the limited resolution of microarrays, combined genomic and epigenomic data extracted from Illumina BeadChip microarrays are a source of stable and reproducible cancer and tissue type specific biomarkers. Furthermore, our results showed that combining omics data with machine learning algorithms and precise data processing strategies can successfully address complex biological challenges, such as pan-cancer classification.

### Funding

OPUS22 grant from National Science Centre, grant ID: 2021/43/B/NZ2/02979.

## A3 Germline variants in cancer patients with personal and family history of colorectal cancer: an update

Asta Försti^1,2,3^

^1^Hopp Children’s Cancer Center (KiTZ), Heidelberg, Germany; ^2^Division of Pediatric Neurooncology, German Cancer Research Center (DKFZ); ^3^German Cancer Consortium (DKTK), Heidelberg, Germany

Multiple primary cancers in a single individual, as well as family history of the same cancer, are features of hereditary cancer. About 15% of colorectal cancer (CRC) patients have first-degree relatives affected by the same malignancy. However, for most families the cause of familial aggregation of CRC is unknown. To identify novel high-to-moderate-penetrance germline variants underlying CRC susceptibility, we performed whole exome sequencing (WES) in germline DNA of Polish CRC patients with personal and family history of colorectal cancer. After WES, we used *in silico* tools followed by protein-protein interaction and functional enrichment analysis to identify most likely rare pathogenic variants predisposing to CRC. In 9 patients sequenced we identified altogether 150 missense, 19 stop_gain, 22 frameshift and 13 canonical splice site variants fulfilling our filtering criteria. The protein-protein interaction analysis of the corresponding genes identified 5 clusters of proteins with enrichment of proteins related to DNA repair, cell cycle, focal adhesion, extracellular matrix interactions and TGFβ signaling pathway. Our findings contribute to the identification of novel genes and pathways as genetic causes of familial CRC.

## A4 Ovarian cancer prevention in the general and in the high risk population

Robert Fruscio^1^

^1^UO Gynecology, Department of Medicine and Surgery, University of Milan-Bicocca, IRCCS, San Gerardo dei Tintori, Monza, Italy

The incidence of ovarian cancer has constantly decreased over the last 30 years, mainly because of the widespread use of oral contraceptives and the increased number of women undergoing prophylactic surgery. Ovarian cancer prevention is currently not possible in the general population, as screening with transvaginal ultrasound and CA125 has been proven to be not effective; on the other hands, in women at high risk of developing ovarian cancer, oophorectomy is safe and reduces the incidence and mortality for ovarian cancer. Since this intervention is associated with the onset of premature iatrogenic menopause, the evaluation of alternative strategies is urgently needed.

## A6 Polygenic inheritance and the risk of ovarian carcinoma

Ewa Grzybowska^1^

^1^Center for Translational Research and Molecular biology of Cancer, Maria Sklodowska-Curie National Research Institute of Oncology, Gliwice branch, Poland

High penetrance mutations in BRCA1 and BRCA2 explain around 25% of the observed familial relative risk and further 10% is explained by moderate risk mutations in MLH1, MSH2, MSH6, RAD51C, RAD51D and B RIP1. Genome-wide association studies (GWAS) have identified ~30 common low-risk SNPs that are associated with ovarian cancer, accounting for approximately 6.5% 0f the FRR. Their inclusion in ovarian carcinoma risk prediction models may improve risk precision. Providing refined personalized cancer risks can result in better risk stratification and hence help in improving early cancer detection and prevention.

In this study we genotyped 225 ovarian cancer patients, 64 breast and ovarian cancer patients and 348 healthy controls. In total, 12 polymorphic variants and 2 deletions in PGR, ABCB1, ABCG2, GSTT1, GSTM1, GSTP1, ATM, TP53 and ATP7B genes were analyzed using ASA-PCR, RFLP-PCR, multiplex-PCR and sequencing. Ten genetic polymorphisms were significantly associated with the risk of developing ovarian carcinoma in at least one of the groups under study. Impact of PGR gene polymorphisms on ovarian cancer risk was specific only for the group of the BRCA1 mutation carriers (in presence of p.Val660Leu variant- OR 2,82; p = 0,010), which confirms the difference in modulation of ovarian cancer risk between sporadic and hereditary malignancies, including the breast-ovarian cancer group (as a cancer-prone group). The analyses showed also the importance ofATP7B gene in ovarian carcinogenesis, both studied variants of which significantly modulated the ovarian cancer risk in all groups excluding the group with BRCA1 mutation. Cumulative risk analysis revealed 3 unfavorable variants that increased significantly the risk of developing ovarian cancer (p.Ile1145 ABCB1+ p.Asp1853Asn ATM+ p.Ser406Ala ATP7B-OR 7,47; p = 0,002) and significantly modified the progression free survival (PFS) of the patients, and also two favorable genotypes which protected against ovarian cancer (p.Arg952LysATP7B+ p.Arg72Pro TP53- OR 0,50; p = 0,008). PFS analysis for carriers of favorable versus unfavorable genotypes emphasized the impact of the regulation of cell cycle(p.Asp1853AsnATM) and active transport of xenobiotics (p.Ser894Ala/ThrABCB1) on the risk of disease progression (HR 3,81; p = 0,010) after paclitaxel/cisplatin chemotherapy. The unfavorable genetic variants could facilitate carcinogenic process and once their carriers developed malignancy, their chances of survival were smaller. Our analyses also showed a strong gene-dosage effect with the decrease of progression-free survival for the carriers of two unfavorable genetic factors.

## A7 Germline genetic of rare cancers

Kari Hemminki^1^

^1^Biomedical Center, Faculty of Medicine, Charles University, 30605 Pilsen, Czech Republic; ^2^Division of Cancer Epidemiology, German Cancer Research Center (DKFZ), Im Neuenheimer Feld 580, D-69120, Heidelberg, Germany

Thanks to panel sequencing, large amounts of data on constitutional variation have recently become available. In the last meetings I have sought to compare these results with population-based family history data. Family studies are able to describe aggregation of any defined cancers in families. The Swedish Family-Cancer Database is the largest of its kind in the world, covering the Swedish families through nearly a century with all cancers in family members since the start of national cancer registration in 1958. The database allows estimation of familial risks, ages of cancer onset and the proportion of familial cancer in different family constellations. However, the focus of comparisons between family and genetic studies has been biased towards the most common cancers for which large volumes of sequencing data has accrued.

Here, I want review, through interactive discussion, rarer solid cancers for which familial risks are often higher than those for common cancers. I want to go through the list of cancers with comments and maybe conclusion if we reach an agreement. Familial risk for lip cancer is 2.0 and it is a rare cancer so familial genetics would not be easy. Small intestinal cancer is also rare but the familial risk is 5.6, higher for the carcinoid component than for adenocarcinoma. Genes for carcinoids have been elusive. Non-medullary thyroid cancer has familial risk of 4.3 and main low-risk genes are known. Thyroid cancer manifests in some rare cancer syndromes including Gardner syndrome (papillary thyroid cancer), familial adenomatous colorectal polyposis (100-fold risk for papillary cancer) and Turcot syndrome (papillary and follicular cancer). Thyroid anaplastic cancer is an exquisitely aggressive tumor for which genetic basis remains unknown. Familial bone cancer is extremely rare, familial risk is 6.9. International collaborative efforts would be beneficial to boost germline genetics of rare cancers.

## A8 BRCA1/2 population screening pilot in latvia. Lessons to learn

Arvids Irmejs^1^, Zanda Daneberga^1^, Hildegunn Hoberg-Vetti^2^, Catherin Cathrine Bjorvatn^2^, Ilva Kononova^3^, Emil Syundyukov^3^, Ieva Danovska^3^, Agate Kalcenaua^3^, Martins Mednis^3^, Eva Pildegovica^3^, Edvins Miklasevics^1^, Janis Gardovskis^1^

^1^Institute of Oncology, Riga Stradins University, Riga, Latvia; ^2^Haukeland University Hospital, Bergen, Norway; ^3^Longenesis Ltd, Riga, Latvia

### Introduction

Approximately 80% of *BRCA1/2* positive breast and ovarian cancers are diagnosed at stages II-IV. Despite the availability of modern treatments, the associated morbidity, mortality and costs remain significant. Therefore, the identification of *BRCA1/2* carriers in a presymptomatic stage is the ultimate goal to improve the prognosis for individuals at risk of hereditary breast and ovarian cancer. In many countries family cascade testing is of limited effectivity due to different reasons. The aim of this study is to evaluate the feasibility of implementing a BRCA1/2 population screening program in Latvia.

### Material and methods

The Institute of Oncology Riga Stradins University (RSUIO), in collaboration with private companies, conducted a pilot study utilizing the digital engagement tool, Longenesis Engage. Eligible women who had previously consented to participate in breast cancer risk assessment projects on skrinings.lv were invited via email, as well as through an email campaign initiated by Lindex. They accessed detailed information on a digital platform and provided consent digitally. Participants, females aged 25–59 without a personal cancer history, completed family cancer history assessments and psychological questionnaires (PHQ9 and GAD-7). Participants then downloaded a laboratory referral form to collect a saliva sample at the nearest private laboratory. Samples were sent to RSUIO for comprehensive BRCA1/2 testing. Negative results will be communicated digitally after completing follow-up questionnaires. In case of positive result, person will be contacted by phone and invited to visit clinical geneticist with following enrolment in the surveillance programme.

### Results

Between September 4th and November 7th, 2023, a total of 3 438 invitation emails were sent as part of the research initiative. Of the recipients, 49% (1 686 out of 3 438) opened the email, and within this group, 54% (925 out of 1 686) visited the project’s digital platform to access detailed project information. Of those who visited the platform, 79% (730 out of 925) provided informed consent, with the majority (86%) opting for a digital signature. Until May, 31^st^, 2024 476 saliva samples had been received at the laboratory. This yields an overall response rate of 14% (476 out of 3 438) for the initial phase of the project. 160 samples have been tested for BRCA1 founders so far. In 2/160 cases BRCA1 pathogenic variants 4035del and 5266dup were identified, Mutation detection rate 1:80.

### Conclusions

The findings from this research initiative suggest that BRCA1/2 population screening through digital communication channels is a viable approach. Preliminary results confirm high BRCA1/2 frequency in the population of Latvia. However, refinement of methodology is mandatory to improve the response rates.

The study is supported by internal grants from Riga Stradins University, with additional funding provided by the private company Lindex. In-kind contributions have also been generously provided by the digital platform provider, Longenesis, and E. Gulbja laboratory.

## A9 Evaluation of serum selenium concentration and polymorphisms of genes responsible for selenium metabolism in women with endometrial cancer

Magdalena Janowska^1^, Tomasz Kluz^1,2^

^1^Department of Gynecology, Gynecology Oncology and Obstetrics, Fryderyk Chopin University Hospital, Rzeszow, Poland; ^2^Institute of Medical Sciences, Medical College of Rzeszow University, Rzeszow, Poland

Numerous studies have shown a relationship between low serum selenium levels and an increased risk of developing cancer. The studies on the influence of selenium level on the development of endometrial cancer are not clear. Numerous studies indicate a relationship between the presence of *GPX1*(rs1050450), *DIO2*(rs225014) and *SEPP1*(rs7579) gene polymorphisms and the development of chronic or neoplastic diseases. However, there are no reports on the influence of these polymorphisms on the development of endometrial cancer.

The mean concentration of selenium was lower in patients with endometrial cancer than in healthy controls (60,63 µg/l vs. 78,74 µg/l, respectively). When compared in quartiles a significant association of lower selenium concentration with the incidence of endometrial cancer was recorded. The highest OR was observed in 1st and 2nd quartiles (OR – 17,8, p-value <0,001; medium selenium level 46,95 µg/l, and OR – 5,94; p-value <0,001; medium selenium level 63,60 µg/l, respectively).

There was a 2,06-fold higher risk of developing endometrial cancer in CC homozygotes *DIO2* (rs225014) polymorphism (95%Cl 1,20-3,59, p-value = 0,009) compared to TT homozygotes. There was no correlation between the occurrence of *GPX1*(rs1050450) and *SEPP1*(rs7579) polymorphisms and the endometrial cancer.

Low selenium levels may be associated with endometrial cancer. Patients with low selenium levels should be a candidate group requiring appropriate preventive examinations. Carrier of the *DIO2*(rs225014) polymorphism may predispose to the development of endometrial cancer. Further research confirming this relationship is recommended.

## A10 Personalized nanodiagnostics of prostate cancer. A proposal for repositioning genetic testing on the timeline of the diagnostic and therapeutic process

Jacek Wilkosz^1^, Tadeusz Kałużewski^2,3^, Dariusz Sobieraj^4^, J Kaczmarek^5^, J Szwalski^6^, Michał Bednarek^2^, Agnieszka Morel^2^, Ż Kasprzyk^2^, Agnieszka Gach^3^, Bogdan Kałużewski^2^

^1^2nd Department of Urology, Medical University of Łódź, Łódź, Poland; ^2^R&D Department, Laboratory of Medical Genetics, GENOS Sp. z o.o., Łódź, Poland; ^3^Department of Genetics, Polish Mother’s Memorial Hospital Research Institute, Łódź, Poland; ^4^Department of Urology, Podkarpacki Regional Hospital, Krosno, Poland; ^5^General Surgical Ward, St John of God Hospital in Łódź, Łódź, Poalnd; ^6^CYTOPATH S. A., Histopathology Laboratory, Łódź, Poland

Prostate cancer (PC) was the most commonly diagnosed malignant tumor in men in Poland in 2019 (20.6%) and the third leading cause of cancer-related deaths in men (10.3%), following lung and colorectal cancer. According to the National Cancer Registry, from 1985, when 2,010 new cases of PC were reported, to 2019, when 17,638 new cases were noted, there was a consistent over eightfold increase in PC incidence. Despite advances in both radical and palliative therapies for PC, there has also been a continuous increase in mortality. In 2019, 5,618 deaths from PC were recorded.

Early and comprehensive diagnostics are crucial in determining the treatment approach and the ultimate outcomes for PC. Transrectal prostate biopsy guided by transrectal ultrasound (TRUS) combined with multiparametric magnetic resonance imaging fusion – Bx MRI/US (fusion biopsy) – is an effective method for PC diagnosis (with a detection rate of up to 68.4% in the “fusion part” of the biopsy combined with systematic prostate biopsy). When additional clinical data and parameters from the diagnostic process (such as patient age, pre-cancerous conditions in previous biopsies, digital rectal examination results, total and free PSA levels in blood serum, prostate volume from TRUS, and PSA derivatives such as PSA density (PSAD) and the free-to-total PSA ratio) are considered using the CART classification tree method for determining indications for Bx MRI/US, this diagnostic tool achieves a sensitivity of 94%, specificity of 84%, and diagnostic accuracy of 90%.

Alongside histopathological studies, genetic testing results are becoming a valuable new source of information that allows for the personalization of both the diagnostic and treatment processes. The material most commonly available to geneticists consists of archived paraffin blocks containing tissue used in HP diagnosis. Even the most meticulously conducted HP procedures can affect the quality and quantity of DNA available to geneticists, not to mention the point in the diagnostic and therapeutic process at which this occurs. We present a method for obtaining prostate biopsy specimens that enables the isolation of high-molecular-weight DNA, meeting the qualitative and quantitative criteria necessary for conducting next-generation DNA sequencing (NGS). We obtained 37 biopsy specimens from 13 patients and standardized the isolation process, consistently obtaining high-molecular-weight DNA samples (5.8 µg/ml - 57.2 µg/ml). The samples were obtained from a single biopsy applied to a membrane using the SmartBx device. In four cases, NGS sequencing was performed using DNA samples from six biopsies, achieving very high-quality parameters (mean sequencing depth ~780x). This allowed for the detection of genetic variants even in a small percentage of cells. These studies are preliminary, preceding the formulation of the final objectives for a new research project.

### References

1. National Cancer Registry; https://onkologia.org.pl/
2. Wojciechowska U. et al: Nowotwory złośliwe w Polsce w 2013 roku. Warszawa, Centrum Onkologii – Instytut im. Marii Skłodowskiej-Curie. Krajowy Rejestr Nowotworów; 2014 r.
3. Wilkosz J. et al: Zastosowanie metody drzew klasyfikacyjnych CART (Classification and Regression Tress) w ustalaniu wskazań do przezkroczowej biopsji stercza nadzorowanej obrazowaniem z fuzji wieloparametrycznego magnetycznego rezonansu jądrowego z przezodbytniczą ultrasonografią gruczołu krokowego w diagnostyce raka stercza - analiza 86 własnych przypadków 49 Kongres Naukowy PTU, Katowice 12.06.2019

## A12 SELINA – cancer risk after optimization of selenium and arsenic blood levels in females with inherited predispositions to breast cancer

Jan Lubinski^1,2^, Wojciech Marciniak^2^, Różą Derkacz^2^, Tomasz Huzarski^1,2,3^, Jacek Gronwald^1,2^, Cezary Cybulski^1,2^, Tadeusz Dębniak^1^, Milena Matuszczak^1^, Adam Kiljanczyk^1^, Adam Stachowski^1^, Paulina Piesik P^1^, Wiktoria Krauze^1^, Krzysztof Lubinski^1^, OlgierdAshuryk^1^, Aleksandra Tołoczko-Grabarek^1^, Ewa Stachowska^4^, Wojciech Lubinski^5^, Dariusz Godlewski^6^, AnnaTomczak^6^, Renata Posmyk^7^, Ewa Kilar^8^, D Czajka^6^, Krzysztof Dach^9^, Magdalena Janowska^10^, Andrzej Jasiewicz^10^, Beata Kozak-Klonowska^11^, Tomasz Kluz^10^, Katarzyna Kluza^10^, R Leśniewicz, Monika Siołek^11^, Marek Szwiec^12^, Joanna Tomiczek-Szwiec^12^, Rafał Wiśniowski^13^

^1^International Hereditary Cancer Center, Department of Genetics and Pathology, Pomeranian Medical University in Szczecin, Szczecin, Poland; ^2^Read-Gene SA, Grzepnica, Poland; ^3^Department of Clinical Genetics and Pathology, University of Zielona Góra, Zielona Góra, Poland; ^4^Department of Human Nutrition and Metabolomics, Pomeranian Medical University in Szczecin, Szczecin, Poland; ^5^II Department of Ophthalmology, Pomeranian Medical University, Szczecin, Poland; ^6^Center of Cancer Prevention and Epidemiology OPEN, Poznań, Poland; ^7^Podlasie Medical Center “Genetics”, Genetic Clinic, Bialystok, Poland; ^8^Independent Public Health Care Center, Oncology Clinic, “Kite” Hospital, Świdnica, Poland; ^9^Voivodship Hospital in Łomża, Department of Pathology and Oncological Prevention, Łomża, Poland; ^10^Podkarpackie Oncology Center, Rzeszów, Poland; ^11^Holly Cross Cancer Center, Kielce, Poland; ^12^Opole Oncology Center, Opole, Poland; ^13^Beskid Oncology Center, Bielsko-Biała, Poland

SELINA is a clinical study on influence of selenium blood levels optimization on cancer occurrence in females from families with increased risk of hereditary breast cancers.

7456 women at the age of at least 40 years have been recruited from centers in Poland. According to planned protocol, 60 months after recruitment, decoding was performed. Compliance rate was 84%. Intervention did not cause undesired effects in any arm.

The strongest positive effects were noted in subgroups of BRCA1(-) (without occurrence of Polish founder constitutional mutations): Selenium deficiency (blood level <98µg/l) on supplement Sel-BRCA1® (ethanol solution of sodium selenite), placebo or diet and selenium excess (blood level >108µg/l) on diet. In all above subgroups combined, cancer risk was decreased ~5 times provided both Arsenic and Selenium levels were analysed. The index risk of cancer (IRC) was created of Se level (µg/l) plus As level multiplied by 50. Optimal IRC was 135-145. IRC 134–145 correlated with almost 5-fold decreased risk of cancers – HR 4.95; CI 1.8-13.8; p-0.002.

### Summary

1. Diet/supplement optimization of Se deficiency and/or excess in females with hereditary predisposition to breast cancer are safe.
2. Lowering of cancer risk in females can be achieved basing on combined analyses of blood levels of Se and As.

### Acknowledgements

Putresza E, Gibaszek R

Grant: INNOMED/I/16/NCBR/2014

## A13 Association between blood levels of selenium, zinc and copper and survival in colorectal cancer patients with lynch syndrome

Magdalena Marciniak^1,2^, Wojciech Marciniak^2^, Piotr Baszuk^1,2^, Martyna Feherpataky^1,2^, Sandra Pietrzak^1^, Wiktoria Krauze^1^, Róża Derkacz^2^, Tadeusz Dębniak^1,2^, Cezary Cybulski^1,2^,Tomasz Huzarski^1,2^, Jacek Gronwald^1,2^, Steven A. Narod^3,4^, K Pullela^3^, Bryson Katona^5^, Ping Sun^3^, Jan Lubiński^1,2^

^1^Department of Genetics and Pathology, International Hereditary Cancer Center, Pomeranian Medical University in Szczecin, Szczecin, Poland; ^2^Read-Gene, Grzepnica, Dobra (Szczecińska), Poland; ^3^Women’s College Research Institute, Toronto, Canada; ^4^Dalla Lana School of Public Health, University of Toronto, Toronto, Canada; ^5^Division of Gastroenterology, Department of Medicine, University of Pennsylvania Perelman School of Medicine, Philadelphia, USA

Lynch syndrome (LS) is a hereditary predisposition to colon and other cancers that is caused by germline mutations in one of four mismatch repair (MMR) genes: *MLH1*, *MSH2*, *MSH6* or *PMS2*. The aim of this study was to evaluate the associations between blood levels of Selenium (Se), Zinc (Zn) and Copper (Cu) taken at or after diagnosis and survival in LS patients with colorectal cancer (CRC). Blood levels of Se, Zn and Cu were quantified in 59 CRC patients with a pathogenic variant in MMR genes. Patients were assigned to one of three tertiles for each element, according to the distribution in the entire cohort. Patients were followed for a mean of 5.0 years from diagnosis or blood taken (whichever is last), during which time 16 deaths were recorded. The age- and sex-adjusted hazard ratios (HR) for all-cause mortality in the middle tertile versus the bottom tertile were 0.28 (95% CI - 0.09-0.88; *p* = 0.03) for Se. For Zn, the HR for all-cause mortality in the top and middle tertiles versus the bottom tertile were 0.05 (95% CI 0.01-0.42; p=0.006) and 0.39 (95% CI 0.13-1.15; *p* = 0.08) respectively. In contrast, elevated Cu levels were associated with relatively poor survival. The HR was 0.15 (95% CI 0.04-0.56; *p* = 0.005) for those in the middle tertile compared to the top tertile. Higher blood levels of Se and Zn and lower blood levels of Cu have a beneficial effect on the survival of patients with CRC and LS.

## A14 Blood elements levels and cancer risk in BRCA1 mutation carriers: a prospective study

Milena Matuszczak^1^, Adam Kiljańczyk^1^, Wojciech Marciniak^2^, Róża Derkacz^2^, Klaudia Stempa^1^, Piotr Baszuk^1,2^, Marta Bryśkiewicz^1,2^, Krzysztof Lubiński^1^, Sandra Pietrzak^1^, Tomasz Huzarski^1,2,4^, Jacek Gronwald^1,2^, Cezary Cybulski^1,2^, Tadeusz Dębniak^1^, Marcin Lener^1^, Anna Jakubowska^1^, Marek Szwiec^3^, Magdalena Stawicka-Niełacna^4^, Dariusz Godlewski^5^, Artur Prusaczyk^6^, Andrzej Jasiewicz^7^, Tomasz Kluz^8^, JoannaTomiczek-Szwiec^9^, Ewa Kilar-Kobierzycka^10^, Monika Siołek^11^, Rafał Wiśniowski^12^, Renata Posmyk^13^, Joanna Tretyn-Jarkiewicz^14^, Rodney Scott^15^, Steven A.Narod^16^, Lubiński Jan^1,2^

^1^Department of Genetics and Pathology, International Hereditary Cancer Center, Pomeranian Medical University, Szczecin, Poland; ^2^Read-Gene, Grzepnica, Dobra (Szczecińska), Poland; ^3^Department of Clinical Genetics and Pathology, University of Zielona Góra Zielona Góra, Poland; ^4^Department of Surgery and Oncology, University of Zielona Góra, Zyty 28, 65–046 Zielona Góra, Poland; ^5^OPEN, Poznań, Poland; ^6^Medical and Diagnostic Center, Siedlce, Poland; ^7^Genetic Counseling Center, Subcarpatian Oncological Hospital, Brzozów, Poland; ^8^Department of Gynecology, Gynecology Oncology and Obstetrics, Institute of Medical Sciences, Medical College of Rzeszow University, Rzeszow, Poland; ^9^Department of Histology, Department of Biology and Genetics, Faculty of Medicine, University of Opole, Opole, Poland; ^10^Department of Oncology, District Specialist Hospital, Leśna 27–29 St, 58-100, Świdnica, Poland; ^11^Holycross Cancer Center, Artwińskiego 3 St, 25-734, Kielce, Poland, Poland; ^12^Regional Oncology Hospital, Wyzwolenia 18 St, 43-300, Bielsko Biała, Poland; ^13^Department of Clinical Genetics, Podlaskie Medical Center, Bialystok, Poland; ^14^Non-Public Health Care Centre, Cancer Genetics Laboratory, Toruń, Poland; ^15^Medical Genetics, Hunter Medical Research Institute; Priority Research Centre for Cancer Research, Innovation and Translation, School of Biomedical Sciences and Pharmacy, Faculty of Health and Medicine, University of Newcastle; Pathology North, John Hunter Hospital, King and Auckland Streets, Newcastle Australia; ^16^Women’s College Research Institute, Women’s College Hospital, University of Toronto, Toronto, Canada

Women with BRCA1 mutations face a significantly elevated risk of breast and ovarian cancers, with lifetime risks of approximately 70% and 40%, respectively. Given this high cancer risk, our study aimed to investigate whether modifiable factors, particularly blood elements levels, could influence cancer risk in this population. We prospectively observed 1,204 initially healthy BRCA1 mutation carriers, most of whom were younger than 50 years (mean age 39). During the 7.5-year follow-up, 206 cancer cases were diagnosed, including 143 breast cancers, 38 ovarian cancers, and 25 other malignancies.

Using ICP-MS, we measured the concentrations of 23 trace elements (Ag, As, Br, Ca, Cd, Co, Cs, Cu, Fe, Hg, I, Li, Mg, Mn, Mo, Ni, P, Pb, Se, Sn, Sr, Ti, V, Zn) in the blood of all participants. The cohort was divided into tertiles based on these elements levels. Cox proportional hazards regression was applied to assess the relationship between elements levels and cancer risk.

Our analysis identified several significant findings. High silver levels were associated with a 6.5-fold increased risk of ovarian cancer, while low cobalt levels reduced ovarian cancer risk nearly threefold. Elevated iodine levels posed a significant danger, increasing ovarian cancer risk by 3.7 times. Similarly, high levels of lithium, molybdenum, vanadium, and zinc were linked to varying cancer risks. An additive protective effect was observed when optimizing multiple elements, with silver, cobalt, lithium, iodine, molybdenum, vanadium, and zinc levels showing an eightfold difference in ovarian cancer risk.

In breast cancer, optimal levels of iodine, phosphorus, and lead modestly reduced risk, and a combined effect of lead and molybdenum resulted in a nearly fourfold reduction. The zinc-to-copper ratio also demonstrated a 1.53-fold reduction in the risk of any cancer. Furthermore, lower levels of cadmium, copper, iodine, and lead were associated with improved overall survival, with lead having the strongest impact on all-cause mortality (a fourfold reduction in risk).

In conclusion, trace elements may serve as important markers of cancer risk in BRCA1 mutation carriers, and their optimization could offer a modest reduction in cancer risk. However, preventive surgeries and regular screenings remain the most effective risk-reduction strategies for this high-risk population. Understanding the interactions between trace elements could offer additional insights for risk management.

## A16 Serum copper, zinc, and copper/zinc ratio levels as biomarkers of survival among prostate cancer patients

Sandra Pietrzak^1^, Wojciech Marciniak^2^, Róża Derkacz^2^, Milena Matuszczak^1^, Adam Kiljańczyk^1^, Piotr Baszuk^1,2^, Magdalena Marciniak^1,2^, Marta Bryśkiewicz^1,2^, Andrzej Sikorski^3^, Jacek Gronwald^2^, Marcin Słojewski^3^, Cezary Cybulski^1,2^, Adam Gołąb^3^, Tomasz Huzarski^1,2,4^, Tadeusz Dębniak^1^, Marcin Lener^1^, Anna Jakubowska^1^, Tomasz Kluz^5,6^, Marianna Soroka^7^, Rodney J Scott^8,9,10^, Jan Lubiński^1,2^

^1^Department of Genetics and Pathology, International Hereditary Cancer Center, Pomeranian Medical University in Szczecin, Szczecin, Poland; ^2^Read-Gene, Grzepnica, Dobra (Szczecińska), Poland; ^3^Department of Urology and Urological Oncology, Pomeranian Medical University in Szczecin, Szczecin, Poland; ^4^Department of Clinical Genetics and Pathology, University of Zielona Góra, Poland; ^5^Department of Gynecology, Gynecology Oncology and Obstetrics, Fryderyk Chopin University Hospital No. 1, Rzeszow, Poland; ^6^Institute of Medical Sciences, Medical College of Rzeszow University, Rzeszow, Poland; ^7^Department of Genetics and Genomics, Institute of Biology, University of Szczecin, Szczecin, Poland; ^8^Priority Research Centre for Cancer Research, Innovation and Translation, Hunter Medical Research Institute, New Lambton, Australia; ^9^School of Biomedical Sciences and Pharmacy, Faculty of Health and Medicine, University of Newcastle, Callaghan, Australia; ^10^Division of Molecular Medicine, Pathology North, John Hunter Hospital, New Lambton, Australia

According to the International Agency for Research on Cancer, prostate cancer is the most commonly diagnosed cancer in men and the second most common cause of cancer-related deaths in men. Survival rates are considered quite good, but they could be enhanced by optimizing specific markers. One of these factors is micronutrients, including copper (Cu) and zinc (Zn). The purpose of our study was to evaluate whether serum levels of Cu, Zn, or Cu/Zn ratio could be related to the survival of patients with prostate cancer. A total of 324 histopathologically confirmed prostate cancer patients were included in the study. Blood samples were collected before treatment and serum levels of elements were assessed using inductively coupled plasma mass spectrometry (ICP-MS). The observation period lasted up to sixty months. All study participants were assigned to the quartiles (QI-QIV) based on the distribution of serum levels of Cu, Zn, and the Cu/Zn ratio among the alive patients. Univariable and multivariable COX regression models were used to calculate hazard ratios (HR) for each quartile of Cu, Zn, and Cu/Zn ratio serum levels. A significant association was found between high serum Cu levels and lower overall survival in prostate cancer patients (HR = 3.08; 95% CI: 1.51-6.29; *p* = 0.002). Prostate cancer patients who died because of the prostate cancer itself, high serum Cu levels were also significant related with worse survival (HR = 3.16; 95% CI: 1.27-7.88; *p* = 0.014). A significant association was also found between low serum Zn levels and worse overall survival in patients with prostate cancer (HR = 3.56; 95% CI: 1.65-7.68; *p* = 0.001) and with worse overall survival in cases of prostate cancer-related deaths (HR = 4.69; 95% CI: 1.59-13.8; *p* = 0.005). Furthermore, we observed a significant association between high serum Cu/Zn ratio and lower overall survival in patients with prostate cancer (HR = 5.66; 95% CI: 2.40-13.3; *p* < 0.001). We also found even stronger significant association between high Cu/Zn ratio and worse survival among prostate cancer patients who died from the disease (HR = 21.4; 95% CI: 2.89-159; *p* = 0.003). These results require further research to establish reference levels for Cu, Zn, and the Cu/Zn ratio in different populations, with the aim of optimizing these serum element levels in individuals with prostate cancer.

## A17 Spectrum of the mutations predisposing to occurrence of colorectal polyposis

Andrzej Pławski^1^, Szymon Hryhorowicz^1^, Marta Kaczmarek-Ryś^1^, Natalia Grot^1^, Alicja Kryszczyńska^1^, Marcin Szuman^1^, Iga Dziechciowska^1^, Monika Knaur^1^, Justyn Hoppe-Gołebiewska^1^

^1^Institute of Human Genetics Polish Academy of Sciences, Poznan, Poland

Polyposis colon is a major feature of several syndromes. Hereditary conditions that cause large numbers of intestinal polyps include familial adenomatous polyposis colonis (MIM 175100), familial adenomatous polyposis type 2 (MIM 608456), Peutz-Jeghers syndrome (MIM 175200), juvenile polyposis syndrome (MIM 174900), and PTEN hamartomatous tumor syndromes (PHTS). Polyps that arise from proliferative dysplasia are called adenomatous polyps or adenomas. They are true neoplastic lesions and are precursors to cancer. Hamartomatous polyps arise from abnormal maturation of the mucosa. They are noncancerous and have no malignant potential. In almost 30 years of polyposis research, we have studied just over 1,000 families with polyposis. Mutations were detected in more than 50% of families and were heterogeneous. We present here a summary of these studies in the face of new challenges related to the study of families in which mutations in predisposition genes have not been detected. The research in part was financed by the project of Polish Ministry of Science and Higher Education project number NdS-IISP0139202401

The research in part was financed by state budget under the program of the Minister of Education and Science called “Science for Society II” the project NCN 2013/09/N/NZ5/02505 amount of funding 1 980 000 zł total value of the project 1 980 000 zł.

## A18 Tumour mutational burden using the TSO-500: How does it help in patient management

Rodney J Scott^1^

^1^Division of Molecular Medicine, NSW Health Pathology & The University of Newcastle, Australia

The TSO-500 is a large panel of 523 genes plus 44 fusions that are associated with cancer development or treatment. The aim of Tumour Mutational Burden (TMB) testing is to reveal what actionable changes occur within a tumour and the number of mutations associated with a given tumour.

The TMB is a measure of the number of mutations per megabase, with more than 10 per megabase being deemed high TMB. The TMB score correlates highly with the likelihood of success in treating patients with immune checkpoint inhibitors (ICIs). Included in the assessment of TMB is microsatellite instability, which contributes to the overall TMB status.

The TSO-500 will detect some gene fusions, somatic variants, novel transcripts, single nucleotide variants (SNVs), insertion deletion variants (indels) and copy number variants. Together, this data provides a comprehensive view of the molecular events underpinning tumour growth that can reveal which targeted therapy is most likely to be effective in treatment of malignancy. Furthermore, the TSO-500 provides information about the variant allele frequency which can be used as a guide to determine if the patient is most likely to carry a germline change. When the variant allele frequency is ~50% confirmatory germline testing is requested.

Finally, TMB assessment can aid in the identification of cancers of unknown primary disease and provide limited information about environmental exposures that result in malignancy. How the TSO-500 is performed, and what requirements are needed to provide useful information from this assay will be presented.

## A19 Results of DMSA long-term supplementation in lowering lead blood level

Siwiec Ewa^1^, Lubiński Jan^1,2^

Department of Genetics and Pathology, International Hereditary Cancer Center, Pomeranian Medical University in Szczecin, Szczecin, Poland; ^2^Read-Gene, Grzepnica, Dobra (Szczecińska), Poland

### Introduction

Our previous study showed that DMSA may be used for effectively lower blood lead concentration and does not cause serious adverse effects.

### Aim of the study

Results of DMSA long-term supplementation in lowering lead blood level. Long-term supplementation – at least 3 months of DMSA supplementation within 6 months period.

### Study groups

1. healthy women BRCA1(-) and Pb concentration > 7,5 µg/L, n=15
2. healthy women BRCA1(+) and Pb concentration > 8,0 µg/L, n=7
3. healthy men and Pb concentration > 13,5 µg/L, n=22

### Results

After long-term supplementation the average value of Pb blood concentration in comparison to initial value was lower by 42,3%, 45,83% and 45,35% in BRCA1(-), BRCA1(+) and men group, respectively. In most cases of BRCA1(-) and BRCA1(+) groups the average value of Pb concentration was higher than the target level, although in male group the average value was lower in 50% subjects.

### Conclusions

During long-term period, we observed a decrease in Pb blood concentration after each month of supplementation and an increase in Pb blood level after supplementation free months. Supplementation decreased exposure to high Pb blood levels although this requires further studies to find out if it can lower cancer risk.

## A20 Blood and urinary arsenic levels are associated with methylation of promoters of genes involved in molecular processes related to arsenic toxicity

Katarzyna Ewa Sokolowska^1^, Jacek Antoniewski^1^, Marta Sobalska-Kwapis^2^, Dominik Strapagiel^2^, Wojciech Marciniak^3,4^, Tomasz Huzarski^3,4^, Jan Lubiński^3,4^, Tomasz Kazimierz Wojdacz^1^

^1^Independent Clinical Epigenetics Laboratory, Pomeranian Medical University,Szczecin, Poland; ^2^Biobank Laboratory, Departament of Oncobiology and Epigenetics, Faculty of Biology and Environmental Protection, University of Lodz, Lodz, Poland; ^3^Department of Genetics and Pathology, Pomeranian Medical University, Szczecin, Poland; ^4^Read-Gene SA, Grzepnica, Poland

### Background

World Health Organization lists arsenic (As) as human toxicant and group one carcinogen. Chronic exposure to As increases the risk of numerous health conditions, including skin lesions, impaired intellectual function, cardiovascular disease, diabetes, inflammation, and cancers including bladder, lung, kidney, liver, skin, and possibly prostate (1, 2). Previous studies have shown that As exposure is associated with changes of both global DNA methylation levels and gene specific methylation. Nevertheless, the molecular mechanism of As exposure related dysregulation of the epigenome are still to be elucidated.

### Methods

Our study included 56 cancer free women from West Pomerania region of Poland with total arsenic concentrations in blood in the range of 0.22-5.77 μg/L and 34 women diagnosed with breast cancer and blood As levels 0.65-4.31 μg/L. The As was measured with ICP-MS (Elan DRC-e, PerkinElmer, USA) and DNA was extracted from diagnostic blood samples using the salting-out method. Genome-wide DNA methylation profiling was performed using the Infinium MethylationEPIC array (Illumina). Raw data were processed with the ChAMP R package. Methylation values were logit-transformed to M-values for robust linear association analysis (limma R package), adjusted for age and cell fraction proportions (calculated based on methylation data). Statistical significance was determined with FDR-corrected p-values, with a cutoff set at 10e-8 and 0.05. The physiological significance of the identified methylation changes was approximated using annotations of regions marked with histones modifications in 11 cell types from 15 state core model. We also conducted GSEA using the FUMA platform and “GO biological processes” and “Hallmark gene sets” databases. We validated our results performing identical analyses on methylation profiling data from 38 cord blood samples for which maternal urinary arsenic was measured and was in the range 6.18-319.74 μg/L.

### Results

Our initial analysis identified 2,453 CpG sites associated (1,794 DMPs positively and 659 negatively) with blood arsenic levels (0.22-5.77 μg/L). The identified CpGs very specifically mapped to the genomic regions marked by histones occupying transcription start sites and gene bodies of actively transcribed genes. The gene ontology terms analyses linked these methylation changes to the physiological processes that have previously been shown to be affected by As exposure such as: cell cycle, mitosis, G2M checkpoint, chromatin organization, or mitotic spindle. Identical analyses performed for the cohort of women diagnosed with cancer and cord blood samples linked CpG sites associated with As levels in these cohorts to the same genomic regions and similar molecular processes.

### Conclusions

Our study performed in three cohorts of individuals exposed to arsenic, uniformly showed that arsenic exposure induces methylation changes specifically at promotors and bodies of actively transcribed genes involved in key molecular processes previously associated with As toxicity. The presence of those changes in blood cells of women exposed to As who developed cancer as well as cord blood, not only validates our results but also suggests that those changes may contribute to the increased risk of cancer observed in exposed individuals.

### Funding

National Science Center, Poland (PRELUDIUM BIS 2: 2020/39/O/NZ2/02943; OPUS 22: 2021/43/B/NZ2/02979)

### References

1. IARC Working Group on the Evaluation of Carcinogenic Risks to Humans. Arsenic, metals, fibres, and dusts. IARC Monogr Eval Carcinog Risks Hum. 2012;100(Pt C):11-465.
2. Moon KA, Oberoi S, Barchowsky A, et al. A dose-response meta-analysis of chronic arsenic exposure and incident cardiovascular disease [published correction appears in Int J Epidemiol. 2018 Jun 1;47(3):1013. doi: 10.1093/ije/dyy073]. Int J Epidemiol. 2017;46(6):1924-1939. doi:10.1093/ije/dyx202

## A21 Blood cells of *BRCA1* mutation and epimutation carriers appear to acquire specific epigenetic signatures

Katarzyna Ewa Sokolowska1, Jacek Antoniewski^1^, Marta Sobalska-Kwapis^2^, Dominik Strapagiel^2^, Tomasz Huzarski^3,4^, Jan Lubiński^3,4,^Tomasz Kazimierz Wojdacz^1^

^1^Independent Clinical Epigenetics Laboratory, Pomeranian Medical University,Szczecin, Poland; ^2^Biobank Laboratory, Departament of Oncobiology and Epigenetics, Faculty of Biology and Environmental Protection, University of Lodz, Lodz, Poland; ^3^Department of Genetics and Pathology, Pomeranian Medical University, Szczecin, Poland; ^4^Read-Gene SA, Grzepnica, Poland

### Introduction

In the population of Polish women, germline mutations in *BRCA1* gene are present in up to 5% of breast cancer cases and the contribution of these mutations to increased breast cancer risk has been extensively studied. Others and us have repeatedly shown, that detectable in blood *BRCA1* promoter methylation (epimutation) is also associated with elevated risk of breast cancer, especially triple-negative breast cancer (TNBC). However, whether these lesion impact genome-wide methylation patterns (methylomes) in the somatic cells of carriers has not been studied so far.

### Methods

We compared methylomes (InfiniumMethylationEPIC microarray data) of blood cells from germline mutation and epimutation carriers and blood cells of individuals sampled 4.7 years prior breast cancer diagnosis, who neither carried the mutation nor the epimutation, to the methylomes of controls confirmed to be cancer-free during an over eight-year follow up period and did not carry either the mutation or the epimutation. We then performed Gene Set Enrichment Analysis (GSEA) to analyze physiological context of the identified methylation changes. As well as assessed the enrichment of histones with specific modifications at the genomic regions harboring *BRCA1* epimutation and germline mutations associated methylation changes. Additionally, we evaluated the presence of these methylation changes in methylomes of TNBC cases, both with and without *BRCA1* mutation and epimutation.

### Results

Our analyses revealed specific methylation signatures in the blood cells of *BRCA1* mutation and epimutation carriers that were absent in the blood of cancer-free women and in the blood cells of women sampled years before diagnosis. The GSEA linked these identified methylation changes not only to the physiological processes but also to genomic regions that have been previously shown to be involved in breast cancer pathology. Most interestingly, an unsupervised clustering analysis confirmed the presence of these changes in methylomes of TNBC cases with somatic *BRCA1* mutation or epimutation.

### Conclusions

Carriers of *BRCA1* mutation and epimutation exhibit distinct genome-wide methylation signatures, that impact specific regions of the genome and biological processes, disruption of which, has previously been shown to contribute to breast cancer pathology. Interestingly, these methylation signatures are absent in the blood cells of breast cancer patients before diagnosis but are detectable in methylomes of TNBC.

### Funding

National Science Center, Poland (PRELUDIUM BIS 2: 2020/39/O/NZ2/02943; OPUS 22: 2021/43/B/NZ2/02979)

## A22 Are current Polish guidelines for prophylactic mastectomy sufficient?

Adam Stachowski^1^, Cezary Cybulski^1^, Jacek Gronwald^1^, Tomasz Huzarski^1^, Tadeusz Dębniak^1^, Milena Matuszczak^1^, Adam Kiljańczyk^]^, Marek Szwiec^2^, Joanna Tomiczek-Szwiec^3^, Andrzej Jasiewicz^4^, Tomasz Kluz^5^, Artur Prusaczyk^6^, Ewa Kilar-Kobierzycka^7^, Dariusz Godlewski^8^, Wojciech Zegarski^9^, Siołek Monika^10^, Beata Kozak-Klonowska^10^, Rafał Wiśniowski^11^, Renata Posmyk^12^, Jan Lubiński^1^

^1^International Hereditary Cancer Center, Department of Genetics and Pathology, Pomeranian Medical University in Szczecin, Szczecin, Poland; ^2^Department of Surgery and Oncology, University of Zielona Góra, Zielona Góra, Poland; ^3^Department of Histology, Department of Biology and Genetics, Faculty of Medicine, University of Opole, Opole, Poland; ^4^Genetic Counseling Center, Subcarpatian Oncological Hospital, Brzozów, Poland; ^5^Department of Gynecology, Gynecology Oncology and Obstetrics, Institute of Medical Sciences, Medical College, Rzeszow University, Rzeszow, Poland; ^6^Medical and Diagnostic Center, Niklinowa 9, 08–110 Siedlce, Poland; ^7^Department of Oncology, District Specialist Hospital, Poland. ^8^OPEN, Poznań, Poland; ^9^Department of Oncological Surgery, Oncology Center in Bydgoszcz, Medical College in Bydgoszcz, Nicolaus Copernicus University in Toruń, Poland; ^10^Holycross Cancer Center, Kielce, Poland; ^11^Regional Oncology Hospital, Bielsko Biała, Poland; ^12^Department of Clinical Genetics, Podlaskie Medical Center, 15–399 Bialystok, Poland

Currently, the Polish Ministry of Health covers the costs of prophylactic mastectomy only for BRCA1/2 mutation carriers or women from families with aggregation of breast cancer. However, these regulations are outdated and should be changed to make the above-mentioned procedure available to other women at high risk of developing breast cancer. Therefore, we suggest covering the costs of mastectomy for carriers of TP53, PTEN, PALB2 mutations regardless of breast cancer family history. In the case of CHEK2 mutations, we suggest limiting access only to women with high-risk variants. These include primarily carriers of protein-shortening variants with a positive family history. We estimated the number of additional prophylactic mastectomies if the Guidelines were changed as proposed by us to be 450.

## A23 PALB2 mutation female carriers registry

Adam Stachowski^1^, Cezary Cybulski^1^, Jacek Gronwald^1^, Tomasz Huzarski^1^, Tadeusz Dębniak^1^, Janusz Jesionka^1^, Joanna Walczyńska^1^, Aniruddh Kashyap^1^, Anna Jakubowska^1^, Marcin Lener^1^, Dominika Wokołorczyk^1^, Wojciech Kluźniak^1^, Marek Szwiec^2^, Joanna Tomiczek-Szwiec^3^, Andrzej Jasiewicz^4^, Tomasz Kluz^5^, Artur Prusaczyk^6^, Ewa Kilar-Kobierzycka^7^, Dariusz Godlewski^8^, Dominik Czajka^8^, Monika Siołek^10^, Beata Kozak-Klonowska^10^, Wiśniowski Rafał^11^, Renata Posmyk^12^, Tomasz Byrski^1^, Małgorzata Stawicka – Niełacna^13^, Jacek Laskus^14^, Paweł Domagała^15^, Krzysztof Dach^16^, Hanna Romanowicz^17^, Christianne Hoey^17^, Steven Narod^17^, Jan Lubiński^1^

^1^International Hereditary Cancer Center, Department of Genetics and Pathology, Pomeranian Medical University in Szczecin, Szczecin, Poland; ^2^Department of Surgery and Oncology, University of Zielona Góra, Zielona Góra, Poland; ^3^Department of Histology, Department of Biology and Genetics, Faculty of Medicine, University of Opole, Poland; ^4^Genetic Counseling Center, Subcarpatian Oncological Hospital, Brzozów, Poland; ^5^Department of Gynecology, Gynecology Oncology and Obstetrics, Institute of Medical Sciences, Medical College, Rzeszów University, Poland; ^6^Medical and Diagnostic Center, Siedlce, Poland; ^7^Department of Oncology, District Specialist Hospital, Świdnica, Poland; ^8^OPEN, Kazimierza Wielkiego, Poznań, Poland; ^9^Department of Oncological Surgery, Oncology Center in Bydgoszcz, Medical College in Bydgoszcz, Nicolaus Copernicus University, Poland; ^10^Holycross Cancer Center, Kielce, Poland; ^11^Regional Oncology Hospital, Bielsko Biała, Poland; ^12^Department of Clinical Genetics, Podlaskie Medical Center, Bialystok, Poland; ^13^Department of Clinical Genetics and Pathomorphology, Institute of Medical Sciences, University of Zielona Góra, Poland; ^14^Salus Health Center, Słupsk, Poland; ^15^Department of Pathology, University Clinical Hospital No 2, Szczecin, Poland; ^16^Private Health Care Facility Department of Pathomorphology and Oncological Prevention, Łomża, Poland; ^17^Department of Pathology, Polish Mother’s Memorial Hospital, Łódź, Poland; ^18^Women’s College Research Institute, Toronto, Canada

Despite the fact that PALB2 mutation carriers are at high risk for aggressive breast cancer, we lack data on optimal treatment methods. The risk factors for the disease in this group remain unknown, let alone non-surgical prevention. The Hereditary Cancer Center in Szczecin has been struggling for 10 years to create a cohort that would be large enough to perform a statistically significant analysis. In this presentation, we would like to show our progress and present you with some interim analyses.

## A25 Genomic instability, microenvironment and telomere homeostasis in solid malignancies

Pavel Vodička^1,2,3^, Michal Kroupa^1, 3^Kristyna Tomasova^1,3^, Anna Siskova^1,2^, Anusha Uttarilli^1^, Saba Selvi^1^, Petr Hanak^1^, Katerina Balounova^1^, Veronika Vymetalkova^1,2,3^, Sona Vodenkova^1,3^, Rajiv Kumar^1^, Kari Hemminki^3^ and Ludmila Vodickova^1,2,3^

^1^Institute of Experimental Medicine, Czech Academy of Sciences, Prague, Czech Republic; ^2^Institute of Biology and Medical Genetics, First Faculty of Medicine, Charles University, Prague, Czech Republic; ^3^Biomedical Center, Faculty of Medicine in Pilsen, Charles University, Pilsen, Czech Republic

Both impaired DNA repair mechanisms and disrupted telomere length homeostasis represent key culprits in cancer initiation, progression and prognosis. Understanding the mechanisms and dynamics of tumor genomic diversification, where DNA damage response and telomere homeostasis are important players, is critical to understand carcinogenesis and overcome the drug resistance. Telomere shortening has a dual role in tumorigenesis. It promotes cancer initiation by inducing CIN, while TL maintenance characterized by telomerase expression is required for cancer cell proliferation and tumour growth. We undertook a comparison of telomere homeostasis genetics (based on GWAS study) with telomere length (TL) in 7,000 patients with sporadic CRC. We also studied TL as a biomarker for cancer risk, patient therapy response and/or survival.

The mitochondrial dysfunction is linked with DNA repair capacity and compensate for damage by increasing the mitochondrial DNA copy number (mtDNA-CN). We investigated mtDNA-CN in CRC tissues and adjacent mucosa in relation with TL. Moreover, gene expressions within DNA repair pathways were related to the damage of mtDNA. The association of mtDNA damage and gene expressions has been found among 48 genes representing all putative repair pathways in mitochondria. In comparison with adjacent mucosa CRC tumors exhibited *lower* extent of mtDNA damage (P=0.047), but there was *higher* expression of the majority of DNA repair genes. Out of 48 DNA repair genes 14 with the strongest correlation between the expression and mtDNA damage (*LIG3*, *MUTYH*, *RAD50*, *BRCA2*, *BRCA1*, *LIG1*, *PARG*, *NEIL3*, *RFC3*, *POLD3*, *POLD4*, *MRE11*, *NEIL1*, *UNG*) are validated on a larger cohort of patients.

### Acknowledgement

AZV NU21-03-00145, NU21-07-00247, NU21-03-00506. GAČR: 21-27902S, 21-04607X, The project National Institute for Cancer Research (Programme EXCELES, No. LX22NPO5102) Funded by the European Union – Next Generation EU.

